# Effect of minocycline on the left ventricular function following ST-elevation myocardial infarction treated by primary percutaneous coronary intervention

**DOI:** 10.1186/s13063-021-05921-2

**Published:** 2022-02-04

**Authors:** Alireza Nasiri, Akbar Shafiee, Ali Hosseinsabet, Azita Hajhosein Talasaz, Arash Jalali, Mojtaba Salarifar

**Affiliations:** Tehran Heart Center, Cardiovascular Diseases Research Institute, Tehran University of Medical Sciences, Tehran, Iran

**Keywords:** Minocycline, ST-elevation myocardial infarction, Left ventricular function, Cardiac remodeling, 2-dimensional speckle tracking echocardiography

## Abstract

**Background:**

Cardiac remodeling following myocardial infarction is a pathological process. We aimed to examine the effect of early short-term minocycline on the left ventricular function following ST-elevation myocardial infarction treated by the primary percutaneous coronary intervention.

**Methods:**

In this double-blind, randomized controlled trial, data of 73 patients STEMI patients who were candidates for primary PCI were enrolled. Patients were then randomized to receive minocycline 50 mg orally, followed by 50 mg once a day for 5 days or a placebo with the same schedule. Measurement of serum matrix metalloproteinase-9 (MMP-9) and 2-dimensional speckle tracking echocardiography was performed at baseline and between 4 and 6 months after discharge. Then the demographic, clinical, echocardiographic, and angiographic data, as well as the levels of MMP-9, were compared between the study groups.

**Results:**

There was no statistically significant difference between the study groups regarding the baseline characteristics. Serum levels of MMP-9 did not change following the intervention within each group and were not significantly different between the groups after follow-up. In the follow-up echocardiography, we also did not observe any difference between the two groups

**Conclusion:**

In this study, we did not observe any effect of minocycline on cardiac remodeling based on 2-dimensional speckle tracking echocardiography and MMP-9 levels.

**Trial registration:**

Iranian Registry of Clinical Trials IRCT201411188698N15. Registered on 22 June 2015, prospectively.

## Introduction

Cardiac remodeling following acute myocardial infarction (AMI) is a pathological process that is followed by cardiac chamber dilation, hypertrophy, and fibrosis of the non-infarcted myocardium [1]. This phenomenon is frequent when left ventricular dysfunction is present [2]. Later and in the ongoing process of cardiac remodeling, heart failure, exercise intolerance, and an increase in mortality may occur [3]; therefore, this issue demands careful attention.

Myocardial ischemia damages both the myocytes and the surrounding extracellular matrix [4]. This event, in turn, activates the metalloproteinases (MMPs) and the resulting zymogens and ends in the inflammation of the cardiac tissue. The final product of this cascade is the inflammation-associated healing and scarring that is described as cardiac remodeling. It is believed that the early inhibition of MMPs can halt the inflammation process and thereby prevent extracellular matrix degradation and cardiac remodeling [5]. Among the MMPs found in the heart is the MMP-9, a gelatinase that highly elevates during heart failure [6, 7].

In order to prevent MMP activation following AMI, some drugs such as tetracycline family have been used in previous studies [8–10]. Minocycline is a cheap and harmless drug of this family whose effect on cardiac remodeling has been proposed previously [11]. We hypothesized that minocycline, which is another member of the tetracyclines, can also inhibit MMP activity and affect scar formation and cardiac remodeling. Therefore, the purpose of this study was to examine the effect of early short-term minocycline administration on the left ventricular function following ST-elevation myocardial infarction (STEMI) treated by the primary percutaneous coronary intervention (PCI).

## Methods

In this double-blind, randomized controlled trial, STEMI patients who presented to our center and were the candidates for primary PCI at Tehran Heart Center were enrolled. A sample size of 90 individuals was calculated based on the results of the Cerisano et al. study [5]. The inclusion criteria were diagnosis of STEMI, defined as chest pain more than 30 min and less than 12 h with ST-segment elevation at the J point in the electrocardiogram for more than 1 mm in at least two consecutive leads, successful PCI, sinus heart rhythm, and giving and informed consent to take part in the trial. The exclusion criteria were poor view in echocardiography, history of heart failure, previous myocardial infarction or cardiac revascularization, history of any cardiac surgery, recent cardiac electrophysiologic interventions, cardiogenic shock at the time of presentation, history of cardiomyopathy or congenital muscular disease, history of chronic diseases or expected reduced life expectancy (i.e., frailty or malignancy), hepatic or renal failure, left bundle branch block in the ECG, receiving thrombolytic therapy, and moderate to severe mitral regurgitation. Also, the patients who did not tolerate the treatment or developed toxicity were excluded from the study. All patients signed informed consent before enrolment. The study protocol was confirmed by the institutional research board and the Medical Ethics Committee of Tehran University of Medical Sciences. The study protocol conforms to the declaration of Helsinki.

At baseline, demographic, physiologic, and clinical data were collected from all the patients. All the patients were asked about the presence of classic cardiovascular risk factors, including diabetes mellitus, hypertension, dyslipidemia, smoking, and a family history of coronary artery disease.

A venous blood sample was obtained from the patients upon entrance to the catheterization laboratory to measure complete blood cell count, fasting blood sugar, lipid profile, creatinine, cardiac troponin-t (cTnT), and creatine phosphokinase-MB (CK-MB). Moreover, a plasma sample was obtained and kept for the baseline measurement of matrix metalloproteinase-9 (MMP-9), to be measured with the follow-up samples between 4 and 6 months. MMP-9 was measured using the human ELISA method (Biovendor, Czech Republic) via ELISA reader STATFAX 2100. The laboratory staff was blinded to the study protocol.

Patients were then randomized to receive minocycline 50 mg orally, followed by 50 mg once a day for 5 days or a placebo with the same schedule. The first dose was prescribed before the procedure. Randomization was performed using an online random number generator software. Then the patients underwent primary PCI according to current guidelines and received all necessary care.

Transthoracic echocardiography was performed in the left lateral decubitus position via the commercial setting (Samsung Medison, Seoul, South Korea), 2–4 MHz probe by an experienced echocardiologist who was blinded to the laboratory data and grouping of the patients. Frist echocardiography was performed within the hospital stay, and the second (follow-up) echocardiography was carried out between 4 and 6 months after hospital discharge. The septum, posterior wall, and left ventricular end-systolic and diastolic diameter were measured in the parasternal long-axis view according to the American Society of Echocardiography (ASE) recommendations. LV end-diastolic and end-systolic volume were measured according to biplane modified Simpson's method in apical four chambers and two chambers’ view. Mitral flow waves (E and A) and deceleration time of E wave were obtained and measured according to the ASE recommendations. Also, early diastolic peak velocity (e′) of septal and lateral mitral annulus were obtained by pulse wave tissue Doppler and the average of these values was computed and reported. Moreover, E/e′ (average) was calculated.

For the two-dimensional speckle tracking echocardiography, three cardiac cycles at the end of expiration were obtained from apical three chambers, apical two chambers, and apical view, with a frame rate of 40–80 f/s and stored in the setting for further analysis. At the end of the systole that was automatically determined with software, the endocardial border was traced from one side of the mitral annulus to another side of it; then, the epicardial border was traced automatically by the software. The following of the endocardial and epicardial border from the traced line was checked. If there was unfollowing of these borders, these stages were repeated. Each wall of the left ventricle was divided by the software into three segments. Myocardial segments with poor signal quality after several trials were excluded. The strain and strain curve of the segments were individually analyzed, and then the average value of the accepted segments was reported. Overall, 17 segments per patient were analyzed.

Between 4 and 6 months from the procedure, all the patients were invited by phone call for the follow-up visit. In the follow-up visit, patients underwent echocardiography with a similar protocol to the baseline, and a blood sample was obtained to measure MMP-9.

Finally, the gathered data were compared between the intervention and the control group, and changes of echocardiographic parameters and MMP-9 levels were compared within and between the groups.

### Statistical analysis

Continuous variables were described with mean and standard deviation (SD) or with median, 25th, and 75th percentiles for skewed data and were compared between coronary artery disease (CAD) and non-CAD groups using Student’s *t* or Mann-Whitney *U* test where appropriate. Categorical variables were expressed as frequency and percentage and among the groups using the chi-square test.

*P* values less than 0.05 were considered statistically significant. The statistical analysis was performed using IBM SPSS statistics for Windows version 22.0 (Armonk, NY: IBM Corp.).

## Results

**Fig. 1:**
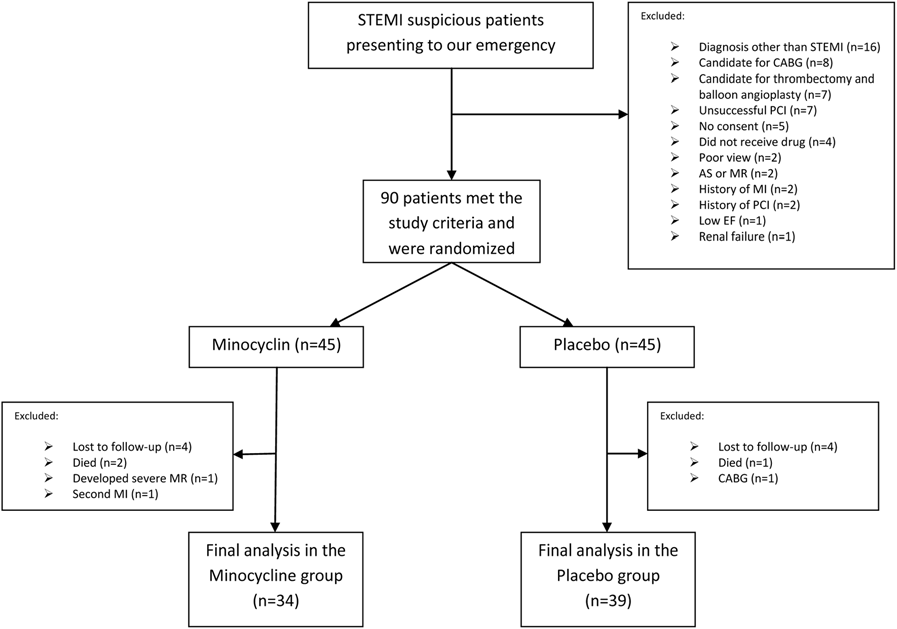
Flow diagram of the study

Within the 8-month study period (March 2015 to November 2015), 147 patients with STEMI presented to the emergency department of Tehran Heart Center. Fifty-seven patients did not meet our criteria, and finally, 90 patients were randomized. We could not complete the follow-up of 17 patients due to the reasons explained in Fig. 1, and finally, 73 patients were included in our final analysis. All patients tolerated the treatment.

There was no significant difference between the study groups regarding the baseline characteristics (Table 1). Similarly, the baseline hemodynamic and echocardiographic characteristics of the study population were not significantly different between the intervention and the control groups, except for diastolic blood pressure, as shown in Table 2.

**Table 1:** Baseline characteristics of the study groups

Characteristic	Control (*n*=39)	Intervention (*n*=34)	*P* value*
Age, year	57.3±9.1	56.5±10.7	0.749
Male gender, *n* (%)	32 (82.1)	31 (91.2)	0.321
BMI, kg/m^2^	27.5±4.3	28.1±2.9	0.510
Diabetes mellitus, *n* (%)	4 (10.3)	6 (17.6)	0.499
Hypertension, *n* (%)	18 (46.2)	10 (29.4)	0.142
Dyslipidemia, *n* (%)	14 (35.9)	20 (58.8)	0.050
Smoking, *n* (%)	15 (38.5)	12 (35.3)	0.780
Family history of CAD, *n* (%)	5 (12.8)	4 (11.8)	0.999
Cholesterol, mg/dl	176.6±47.0	173.4±37.2	0.750
Triglyceride, mg/dl	125.0 [88.0, 155.0]	120.0 [78.5, 161.0]	0.932
LDL, mg/dl	114.0 [84.0, 133.0]	112.0 [88.0, 132.5]	0.906
HDL, mg/dl	40.0±8.9	39.6±9.5	0.857
Fasting blood sugar, mg/dl	110.0 [98.0, 126.0]	119.5 [99.0, 137.0]	0.210
Creatinine, mg/dl	0.88±0.17	0.90±0.19	0.645
MMP-9, μg/dl	111.0±20.0	106.5±27.4	0.437

*BMI* body mass index, *bpm* beats per minute, *CAD* coronary artery disease, *HDL* high-density lipoprotein, *LDL* low-density lipoprotein, *MMP* matrix metalloproteinase

*P value less than 0.05 was considered as statistically significant

**Table 2:** Comparing the baseline echocardiographic characteristics between the study groups

Characteristic	Control (*n*=39)	Intervention (*n*=34)	*P* value
Systolic blood pressure, mmHg	117.8±17.4	119.1±13.1	0.735
Diastolic blood pressure, mmHg	72.5 [66.2, 80.5]	76.0 [70.0, 80.0]	0.357
Heart rate, bpm	77±12	72±15	0.144
LVDd	51.6±5.1	49.4±3.8	0.042
LVDs	37.3±6.3	36.5±40.0	0.516
PW	9.2±1.4	9.2±1.6	0.919
IVS	9.3±1.4	9.5±1.7	0.761
LVEDV	86.8±22.2	87.8±22.2	0.852
LVESV	49.3±18.7	49.4±14.7	0.981
LVEDV index	46.1±11.3	44.7±9.6	0.574
LVESV index	26.3±10.0	25.6±7.4	0.642
EF Simpson	44.2±10.8	43.9±8.0	0.870
PEV	69.2±19.2	60.9±16.2	0.065
PAV	70.3±17.0	64.7±18.8	0.211
E/A	1.04±0.38	1.02±0.41	0.834
Deceleration time	193.6±60.7	199.8±63.9	0.688
e septal	5.8±1.4	6.1±1.5	0.467
e lateral	6.8±2.1	7.7±2.2	0.092
E/e′	11.3±3.7	9.1±2.8	0.009
Global systolic strain	− 11.8±3.3	− 11.5±3.0	0.654
SRs	− 1.0±0.3	− 1.0±0.4	0.955
Sre	1.0±0.4	1.0±0.2	0.507
Sra	0.7±0.3	0.7±0.2	0.398

*bpm* beats per minute, *IVS* interventricular septum, *LVDd* left ventricular diastolic diameter, *LVDs* left ventricular systolic diameter, *PAV* peak A velocity, *PEV* peak E velocity, *PW* posterior wall, *SR* strain rate

*P value less than 0.05 was considered as statistically significant

Serum levels of MMP-9 did not change following the intervention within each group and were not significantly different between the groups both at baseline (*P*=0.437) and after follow-up (96.6±17.7 μg/dl in the intervention group vs. 101.8±23.1 μg/dl in the controls, *P*=0.283).

In the follow-up echocardiography, we also did not observe any difference between the two groups (Table 3). In the follow-up analysis, the echocardiographic evaluation showed that none of the speckle tracking variables were significantly different between the groups (global strain: *P*=0.997; SRs: *P*=0.965; SRe: *P*=0.684; and SRa: *P*=0.398). We observed no specific drug-related adverse event within the study period in our study population.

**Table 3:** Comparing the follow-up echocardiographic characteristics between the study groups

Characteristic	Control (*n*=39)	Intervention (*n*=34)	*P* value
Systolic blood pressure, mmHg	133.5±24.8	125.9±16.4	0.263
Diastolic blood pressure, mmHg	80.0±10.3	76.0±8.4	0.177
Heart rate, bpm	74.5±14.1	70.7±15.0	0.290
LVDd	50.9±5.6	49.2±5.4	0.191
LVDs	37.2±7.3	35.2±6.7	0.218
PW	8.7±1.2	8.9±1.1	0.541
IVS	9.0±1.2	90.1±1.1	0.743
LVEDV	88.7±22.6	89.8±28.6	0.626
LVESV	48.7±16.2	49.7±21.6	0.844
LVEDV index	46.3±12.1	45.9±13.8	0.888
LVESV index	26.1±9.1	25.4±10.9	0.774
EF Simpson	44.4±8.1	45.9±8.9	0.452
PEV	60.6±16.0	60.0±18.0	0.885
PAV	73.1±20.4	70.0±19.9	0.533
E/A	0.9±0.3	0.9±0.3	0.597
Deceleration time	248.0±80.4	238.5±52.7	0.572
e septal	6.0±1.4	6.2±1.5	0.476
e lateral	7.5±2.9	8.2±2.7	0.300
E/e′	9.5±3.3	8.6±3.1	0.257
Global systolic strain	− 13.0±2.8	− 13.0±3.1	0.997
SRs	− 1.1±0.3	− 1.1±0.2	0.965
Sre	1.09±0.30	1.06±0.30	0.684
Sra	0.7±0.2	0.7±0.2	0.398

*bpm* beats per minute, *IVS* interventricular septum, *LVDd* left ventricular diastolic diameter, *LVDs* left ventricular systolic diameter, *PAV* peak A velocity, *PEV* peak E velocity, *PW* posterior wall, *SR* strain rate

*P value less than 0.05 and as statistically significant

## Discussion

The role of MMP activation in the process of cardiac remodeling is well-established [12]. Although previous studies (mentioned below) have shown the effect of MMP inhibitors on the attenuation of cardiac remodeling following AMI, we failed to show a treatment benefit of minocycline in the remodeling process.

Following sudden myocardial ischemia, both the gene expression and activation of MMP increase in the extracellular matrix and a pathologic cascade of collagen and elastic fiber degradation, structural remodeling of the extracellular matrix, impaired collagen formation, and myocardial fibrosis happens [13–15]. Thus, the termination of this cascade through the inhibition of MMP activation seems logical. One of the drugs that have been successfully used for the inhibition of MMP activation was doxycycline [16]. Both doxycycline and minocycline belong to the tetracycline family of antibiotics. However, it has been proposed that both of them have some other non-antibiotic properties [17]. Additionally, minocycline possesses various advantages, including a good safety profile as an antibiotic as well as being a pharmacoeconomic drug [18]. Consequently, we used minocycline in the present study as a safe and low-cost drug and observed its effect on cardiac remodeling. As far as this was the first study on the effect of minocycline on cardiac remodeling, we selected the lowest possible effective dose based on the levels of MMP and othr markares following STEMI that generally remain elavated for 5 days following infarction.

Unlike doxycycline, which has been shown to improve cardiac remodeling in several studies [5, 9, 19, 20], there are very few studies regarding the use of minocycline for the inhibition of MMP activation and, thereby, cardiac remodeling. One experimental study showed that minocycline could protect the adult rat cardiac myocytes against simulated ischemia-reperfusion injury by inhibiting poly (ADP-ribose) polymerase-1 [21]. In another experimental study, it was observed that minocycline has multiple inhibitory effects on vascular endothelial growth factor-induced smooth muscle cell migration, a process that meddles with normal angiogenesis and vascular remodeling [22]. Following In vivo experimental ischemia and reperfusion on rats, minocycline significantly decreased the infarct size by about 33%, tissue MMP-9 activity and oxidative stress [11] same authors reported that the cardioprotective effect of minocycline occurred when ints concentration in normal myocyte reached 0.5 mmol/L and 1.1 mmol/L in the ischemic myocyte. In a recent experimental study, minocycline could attenuate systemic inflammation and heart failure following AMI in rats [23]. However, in our study, we did not observe any improvement in the cardiac remodeling by minocycline as detected by echocardiography and MMP-9 levels. This finding may result from the fact that all previous studies with minocycline were experimental, and thereby the effect of the drug on human subjects may differ from in-vivo situations. Our finding is comparable to Tessone et al. study [24] that showed long-term doxycycline (up to 10 days after infarction) does not prevent LV remodeling, and on the contrary, it disturbed angiogenesis and increased compensatory LV hypertrophy and remodeling. Similar findings were also shown by Garcia et al. [16]. Therefore, it seems that the effect of these drugs is time-dependent, so the timing of the MMP inhibitor should be carefully considered. We recommend more studies with various protocols of minocycline administration in humans in order to clarify its cytoprotective effects.

### Study limitations

It is essential to note the limitations associated with our study. The limited sample size of this study is our major limitation. On the other hand, we only measured MMP-9; the measurement of other factors such as MMP-3 or interleukins could probably provide more information regarding the use of minocycline in STEMI patients. The limited follow-up period is also another limitation of our study. In a longer follow-up, one could observe the effect of minocycline on exercise capacity, cardiac events, and mortality.

## Conclusion

In this study, we found that early short-term minocycline does not affect the left ventricular function following STEMI, as well as other echocardiographic levels and MMP-9. Due to the established cytoprotective effects of minocycline, we recommend further clinical trials with more extended follow-up periods and measurements of other similar biomarkers to establish knowledge on this topic and define an effective dose.

## Data Availability

Data is available upon request from the corresponding author.
